# Genetic variants in the Folic acid Metabolic Pathway Genes predict outcomes of metastatic Colorectal Cancer patients receiving first-line Chemotherapy

**DOI:** 10.7150/jca.44580

**Published:** 2020-09-21

**Authors:** Lu Jiang, Shuwei Li, Ming Yuan, Ling Ma, Yu Lin, Weiyou Zhu, Haina Du, Meilin Wang, Tao Chen, Lingjun Zhu

**Affiliations:** 1 Department of Oncology, The First Affiliated Hospital of Nanjing Medical University, Nanjing, China.; 2 Jiangsu Key Lab of Cancer Biomarkers, Prevention and Treatment, Jiangsu Collaborative Innovation Center For Cancer Personalized Medicine, Nanjing Medical University, Nanjing 211166, China.; 3 Department of Oncology, Jiangyin People's Hospital, Wuxi, China.; 4 Nanjing Hospital of Chinese Medicine Affiliated to Nanjing University of Chinese Medicine, Nanjing, China.; 5 Department of Gastrointestinal Surgery, The First Affiliated Hospital of Nanjing Medical University, Nanjing, China.

**Keywords:** genetic variants, folic acid, colorectal cancer, chemotherapy

## Abstract

**Background:** The association between genetic variants in the folic acid metabolic pathway genes and survival, as well as the responses to chemotherapy of metastatic colorectal cancer (mCRC) patients has not been reported.

**Methods:** The association between genetic variants in the folic acid metabolic pathway genes and progression-free survival (PFS) and overall survival (OS) of mCRC patients were analyzed using Cox regression model. The false discovery rate (FDR) correction method was conducted. The logistic regression model was used to explore the effects of the interested genetic variants on disease control rate (DCR). The Cancer Genome Atlas (TCGA) database was applied to compare gene expression differences.

**Results:** We found that rs3786362 G allele of thymidylate synthase (*TYMS*) gene was significantly associated with PFS (P = 1.10 × 10^-2^), OS (*P* = 2.50 × 10^-2^) and DCR (*P* = 5.00 × 10^-3^). The expression of *TYMS* was overexpressed in CRC tissues compared with adjacent normal tissues. Furthermore, *TYMS* expression level decreased with respect to younger age and advanced tumor stage.

**Conclusion:** Genetic variants in the folic acid metabolic pathway genes might serve as potential prognostic biomarkers for mCRC patients.

## Introduction

In 2020, there will be 147,950 newly diagnosed colorectal cancer (CRC) patients and 53,200 deaths from the disease in the United States. The morbidity and mortality of CRC among individuals aged younger than 50 years increased during recent years 1. CRC is the third cause of cancer-related death worldwide the fifth cause of cancer-related death in China 2. Patients often have distant metastases at the time of diagnosis 3, leading to an increasing enormous burden.

Folic acid was firstly reported as a fundamental micronutrient in the twentieth century 4. A deficiency in cellular folic acid contributes to aberrant DNA methylation and apoptosis of cancer cells 5. There is evidence that abnormal biosynthesis and metabolism of folic acid are correlated with progression of CRC 6. 5-fluorouracil (5-FU), acting as an anti-folate chemical medicine, has been a cornerstone for the clinical treatment of CRC during the past five decades 7. The standard first-line chemotherapy regimens for metastatic colorectal cancer (mCRC) include FOLFOX (5-FU, folinic acid, oxaliplatin), FOLFIRI (5-FU, folinic acid, irinotecan) and XELOX [capecitabine (a 5-FU prodrug), oxaliplatin] in combination with or without targeted biologics. XELIRI (capecitabine, irinotecan) regimen exhibits similar efficiency in clinical treatment. 5-FU mainly targets at enzymes that were encoded by a series of susceptible genes in the folic acid metabolic pathway such as thymidylate synthase (*TYMS*), methylenetetrahydrofolate reductase (*MTHFR*) and reduced folate carrier 1 (*RFC1*) 7, 8.

Mortality rates of CRC decrease by surgery and chemotherapy treatment 9. However, survival time and treatment responses of mCRC patients undergoing first-line chemotherapy remain inconsistent. Several prognostic factors for CRC were identified by a number of epidemiological studies, such as obesity, tumor site and therapy 10. Moreover, genetic variants in important metabolic pathway genes, such as estrogen metabolic pathway genes and methionine metabolic pathway genes were reported to have effects on CRC prognosis 11, 12. Therefore, the identification of single-nucleotide polymorphisms (SNPs) in complicated gene-phenotype-cancer pathways may provide insights into prognosis prediction for CRC patients. According to Jang, M. J. et.al 13, genetic variants in two key genes in the folic acid metabolic pathway, *TYMS* and *RFC1*, were proved to relate to CRC prognosis in a Korean population. However, few studies explored the association between genetic variants in this pathway and the responses to first-line chemotherapy in mCRC patients. In our study, the systematic evaluation was performed to examine the potential roles of folic acid metabolic pathway SNPs in outcomes (including survival and the responses to chemotherapy) of mCRC patients treated with first-line chemotherapy.

## Materials and Methods

### Study subjects

A total of 325 mCRC patients were retained in our research from the First Affiliated Hospital of Nanjing Medical University and Nanjing First Hospital from September 2010. To be eligible for our analysis, patients had to meet the following criteria: (1) histologically diagnosed with mCRC; (2) receiving first-line chemotherapy regimens including irinotecan-based (FOLFIRI and XELIRI) or oxaliplatin-based (FOLFOX and XELOX) chemotherapy in combination with or without targeted biologics; (3) receiving at least two cycles of chemotherapy before evaluation; (4) undergoing regular examination before and after chemotherapy; (5) having measurable solid lesions scanned by computed tomography (CT) before treatment; (6) unrelated Han Chinese. Patients that suffered from other primary tumors, cardiopulmonary insufficiency or severe infection were excluded. In addition, we excluded mCRC patients with liver metastasis whose alanine aminotransferase (ALT) was higher than 90U/L. Each peripheral blood sample of patients was collected in admission and preserved ethylenediaminetetraacetic acid (EDTA) tubes for DNA extraction. The protocol was approved by the Institutional Review Board of Nanjing Medical University. All patients gave their written informed consent before enrollment and the clinical characteristics of patients were described previously 14.

### The evaluation of clinical outcomes

The outcomes were evaluated by CT before treatment and after a minimum of two cycles of chemotherapy in mCRC patients. The calculated clinical outcomes were the progression-free survival (PFS), overall survival (OS) and responses to chemotherapy. The primary endpoint was PFS assessed on the basis of the Response Evaluation Criteria in Solid Tumors (Version 1.1) 15. CR (complete response), PR (partial response), PD (progress disease) and SD (stable disease) were used to assess the survival and responses to chemotherapy. PFS was defined as time from date of primary chemotherapy treatment to date of diagnosis of PD or to date of last follow-up. OS was defined as time from date of primary chemotherapy treatment to date of death or last recorded date of follow-up. Disease control rate (DCR) was defined as the percentage of patients who achieved CR, PR, or SD. The follow-up duration of all the patients were performed periodically through telephone calls.

### The selection of folic acid metabolic pathway-associated genes and SNPs

The selection of genes in the folic acid metabolic pathway was conducted from the Kyoto Encyclopedia of Genes and Genomes (KEGG) (https://www.kegg.jp/kegg/pathway.html), AmiGO 2 (http://amigo.geneontology.org/amigo/landing) and PubMed (https://www.ncbi.nlm.nih.gov/). Genes that are essential in the folic acid metabolic pathway and have been supported to connect with CRC susceptibility, as well as survival by epidemiologic or experimental data were finally selected for further investigation.

Firstly, SNPs within the candidate genes and 2 kb upstream regions were extracted by the Han Chinese in Beijing (CHB) data from the 1000 Genomes Project (March 2012) with the following criteria for quality control: (a) a call rate ≥ 99%; (b) minor allele frequency (MAF) ≥ 10%; and (c) Hardy-Weinberg Equilibrium (HWE) exact *P* value ≥ 0.05. Secondly, potential functions of SNPs were analyzed based on RegulomeDB (http://regulome.stanf ord.edu/index), HaploReg (http://archive.broadinstitute.org/mammals/haploreg/haploreg. php), GTEx portal (http://www.gtexportal.org/home/) and SNPinfo Web Server (http://snpinfo.niehs.nih.gov/). SNPs above RegulomeDB score 5 were excluded. Thirdly, representative tagging SNPs in low linkage disequilibrium (LD) (*r*^2^ < 0.8) were selected using HaploView 4.2 software. Fourthly, we investigated the association between the candidate SNPs and PFS of mCRC patients with adjustment for sex and age in the additive model. The false discovery rate (FDR) correction was conducted and we selected SNPs of which adjusted *P*_FDR_ (PFS) value was < 0.15. Finally, the effects of the candidate SNPs on DCR were analyzed in the additive model and SNPs statistically related to DCR (adjusted *P* < 0.05) were selected.

### SNP genotyping

The Qiagen Blood Kit (Qiagen) was used to extract genomic DNAs from collected blood samples. Genotyping was performed using Illumina Human Omni ZhongHua Bead Chips. We filtered the samples and SNPs using a uniform quality control protocol.

### Functional Annotation of the selected genes

The Cancer Genome Atlas (TCGA) database (http://cancergenome.nih.gov/) was used to analyze differential gene expression of RNA-sequencing data in CRC tissues and adjacent normal tissues (log2 transformed). In addition, we conducted gene expression analysis in subgroups of age, sex, tumor site, family history, body mass index (BMI) and tumor stage. Survival analysis was performed to search for the relationship between the expression of the selected genes and OS of patients.

### Statistical analysis

In order to eliminate several potential confounders in targeting individuals, unconditional univariate and multivariate Cox regression models were applied to estimate the relationships between patient characteristics and OS, including sex, age, tumor site, tumor grade, number of metastatic organism, drinking status, smoking status, family history, Dukes stage and chemotherapy regimens. Multivariate Cox regression model was conducted to calculate adjusted hazard ratios (HRs) and 95% CIs for the relationship between SNPs and survival of mCRC patients. Multivariate logistic regression model was used to estimate the adjusted odds ratios (ORs) and their 95% CIs for exploring the association between SNPs and DCR. We firstly used multivariate Cox and logistic regression models to explore the correlation between the candidate SNPs and PFS, as well as DCR of mCRC patients with adjustment for sex and age in the additive model. Considering that tumor site and chemotherapy regimens could have potential effects on outcomes of patients, we further conducted multivariate analysis to investigate the association between the interested SNPs and outcomes of mCRC patients with adjustment for sex, age, tumor site and chemotherapy. We applied the FDR correction method for adjusted *P* values to conducting multiple comparisons. Moreover, Kaplan-Meier curves were performed to demonstrate the correlation between the interested SNPs and cumulative survival probability of PFS and OS. Unpaired student *t*-test was applied to compare the different gene expression levels between tumor tissues and normal tissues based on TCGA database. Stratified analysis according to TCGA database was evaluated using ANOVA in subgroups of tumor stage.

All statistical computation was achieved by PLINK (version 1.09) and R software (version 3.2.3). It was considered statistically significant for *P* values < 0.05.

## Results

### Characteristics of the study population

The detailed information regarding the clinical characteristics of 325 patients and their associations with OS is shown in **Supplementary Table S1.** Among these patients, 205 were males (63.1%) and 120 were females (36.9%). All patients were diagnosed with advanced Dukes stage (C or D) cancer after surgery operations, with 194 colon cancer and 131 rectal cancer patients. At the end of follow-up, 188 patients received oxaliplatin-based chemotherapy with 81 (43.1%) deaths, and 131 patients received irinotecan-based chemotherapy with 69 (50.4%) deaths. In summary, no clinical characteristics of patients were considered as confounders in our study.

### The selection of genes and SNPs in the folic acid metabolic pathway

**Figure 1:**
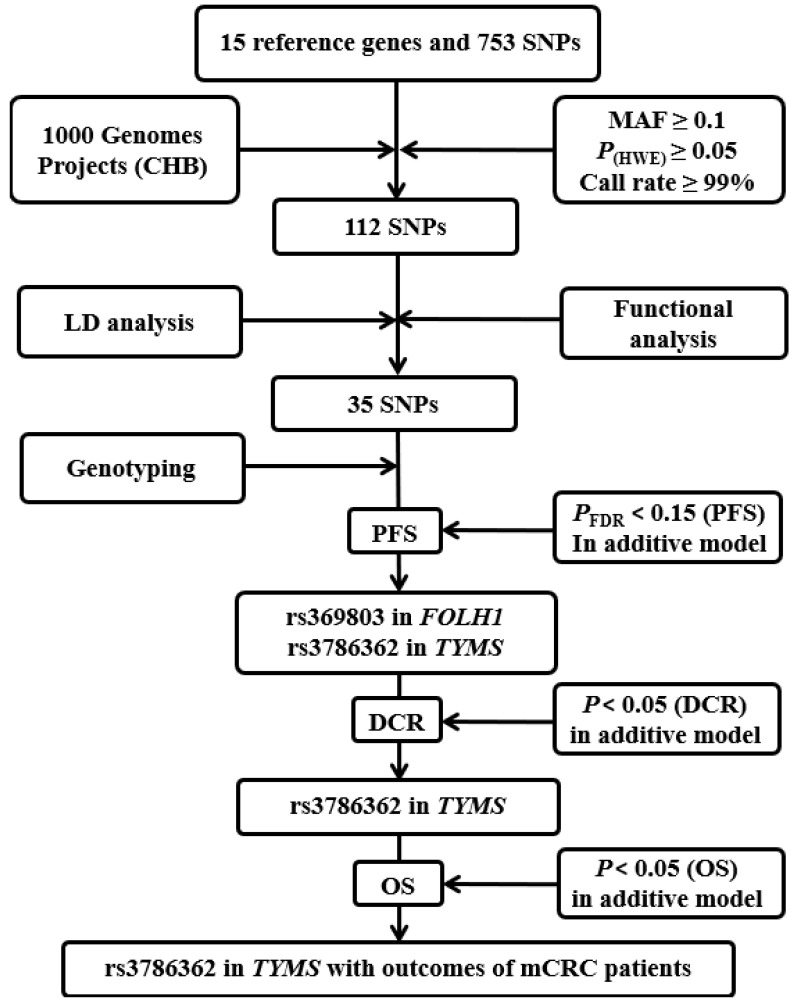
** Flow chart for selecting SNPs in the folic acid metabolic pathway genes.** CHB: the Han Chinese in Beijing; MAF: minor allele frequency; HWE: Hardy-Weinberg Equilibrium; LD: linkage disequilibrium; PFS: progression-free survival; DCR: disease control rate.

The detailed progress of selecting genes and SNPs in the folic acid metabolic pathway is exhibited in **Figure 1.** Fifteen key genes were selected for further study after the thoroughly extraction from KEGG, AmiGO 2 and published studies. The detailed information of 15 key genes is shown in** Supplementary Figure S1** and **Supplementary Table S2**.

We firstly identified a total of 753 SNPs that were located in 15 candidate gene regions, including 2 kb upstream. Only 112 SNPs remained after quality control. After conducting functional analysis and LD analysis, 35 putative functional SNPs were retained and the detailed information of 35 SNPs for function annotation after silico analysis is listed in **Supplementary Table S3**.

### The association between 35 SNPs and PFS of mCRC patients

We analyzed the association between 35 SNPs and PFS of mCRC patients in the additive model after genotyping (**Supplementary Table S4**). As shown in **Table 1**, we found that four SNPs (rs369803 in *FOLH1*, rs10432965 in *FTCD*, rs4795436 in *SLC46A1* and rs3786362 in *TYMS*) were correlated with PFS. After FDR correction, only two SNPs (rs3786362 in *TYMS* and rs369803 in *FOLH1*) were significantly associated with PFS of mCRC patients (rs3786362: HR = 1.43, 95% CI = 1.12-1.82, *P*_FDR_ = 0.10; rs369803: HR = 0.68, 95% CI = 0.51-0.89, *P*_FDR_ = 0.10). Then, we evaluated the correlation of two candidate SNPs with DCR as demonstrated in **Table 1**. Interestingly, only *TYMS* rs3786362 was both related to reduced PFS and DCR (OR = 1.97, 95% CI = 1.19-3.27, *P* = 8.00 × 10^-3^) after adjustment for sex and age in the additive model. Consequently, *TYMS* rs3786362 was selected for subsequent analysis. With adjustment for sex, age, tumor site and chemotherapy, rs3786362 in *TYMS* was observed to correlate with reduced PFS (HR = 1.37, 95% CI = 1.08-1.75, *P* = 1.10 × 10^-2^) and DCR (OR = 2.07, 95% CI = 1.24-3.44, *P* = 5.00 × 10^-3^) of mCRC patients in the additive model, which is consistent with the results mentioned above (**Table 2 and Table 3**).

### The correlation between rs3786362 in *TYMS* and mCRC survival

Owing to the positive findings of *TYMS* rs3786362 on PFS in our previous study, the correlation analysis of the selected SNP with OS was further conducted. Interestingly, *TYMS* rs3786362 was also associated with reduced OS in the additive model (HR = 1.43, 95% CI = 1.03-1.85, *P* = 1.90 × 10^-2^) (**Table 2**). In addition, it was found that AG genotype of rs3786362 in TYMS was both correlated with reduced PFS (HR = 1.65, 95% CI = 1.24-2.19, *P* = 1.00 × 10^-3^) and OS (HR = 1.54, 95% CI = 1.09-2.18, *P* = 1.00 × 10^-2^) compared with AA genotype. In subsequent SNP-associated analysis with additive, dominant and recessive models, carriers of rs3786362 G allele were prone to have shorter PFS and OS time in the dominant model (PFS: HR = 1.55, 95% CI = 1.17-2.05, *P* = 2.00 × 10^-3^; OS: HR = 1.55, 95% CI = 1.10-2.18, *P* = 1.20 × 10^-2^) with adjustment for sex, age, tumor site and chemotherapy in multivariate analysis. However, no significant differences were observed in the recessive model.

**Figure 2:**
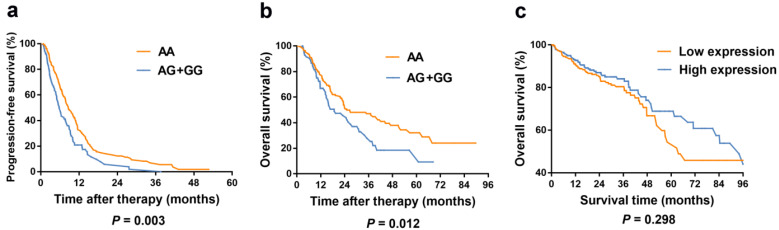
** Kaplan-Meier curves in colorectal cancer patients according to our study and TCGA database.** (**a**) Kaplan-Meier curves of progression-free survival for rs3786362 in mCRC patients using Cox regression model. (**b**) Kaplan-Meier curves of overall survival for rs3786362 in mCRC patients using Cox regression model. (**c**) Kaplan-Meier curves of survival rate for *TYMS* expression levels in colorectal cancer patients according to TCGA database. HR: hazard ratio; CI: confidence interval.

Kaplan-Meier curves of PFS and OS for *TYMS* rs3786362 in mCRC patients were depicted choosing the dominant model. Patients with AG/GG genotypes exhibited reduced PFS and OS compared with AA genotype (**Figure 2**).

### The correlation between rs3786362 in *TYMS* and DCR of mCRC patients

In order to explore the correlation of rs3786362 in *TYMS* and responses to first-line chemotherapy in mCRC patients, we conducted three models including additive model, dominant model and recessive model for correlation analysis. Our study discovered that rs3786362 G allele was associated with reduced DCR both in the additive model (OR = 2.07, 95% CI = 1.24-3.44, *P* = 5.00 × 10^-3^) and dominant model (OR = 2.44, 95% CI = 1.38-4.30, *P* = 2.00 × 10^-3^) after adjusting for sex, age, tumor site and chemotherapy, suggesting a considerable effect of the G allele on DCR (**Table 3**).

### Stratification analysis of rs3786362 in *TYMS* and mCRC survival

Stratification analysis was also performed to evaluate the potential effects of *TYMS* rs3786362 in mCRC patients in the dominant model. Overall, the carriers of the risk G allele reduced PFS with respect to female, younger age, colon cancer, well and moderate tumor differentiation, with metastatic organism ≤ 2, drinking status, and non-smoking status, no CRC family history and recipients of oxaliplatin-based chemotherapy in multivariate analysis (**Table 4**). The OS time was shorter for patients with AG/GG genotype in subgroups of male, younger age, rectal cancer, well and moderate tumor differentiation, with metastatic organism ≤ 2, non-drinking status, non-smoking status and recipients of irinotecan-based chemotherapy (**Table 4**). We then hypothesized that *TYMS* rs3786362 might have important effects on PFS and OS in mCRC patients and could be a predictive biomarker for survival of mCRC patients in subgroups of younger age, well and moderate tumor differentiation, with metastatic organism ≤ 2 and non-smoking status.

### *TYMS* expression differences and survival analysis based on TCGA database

**Figure 3:**
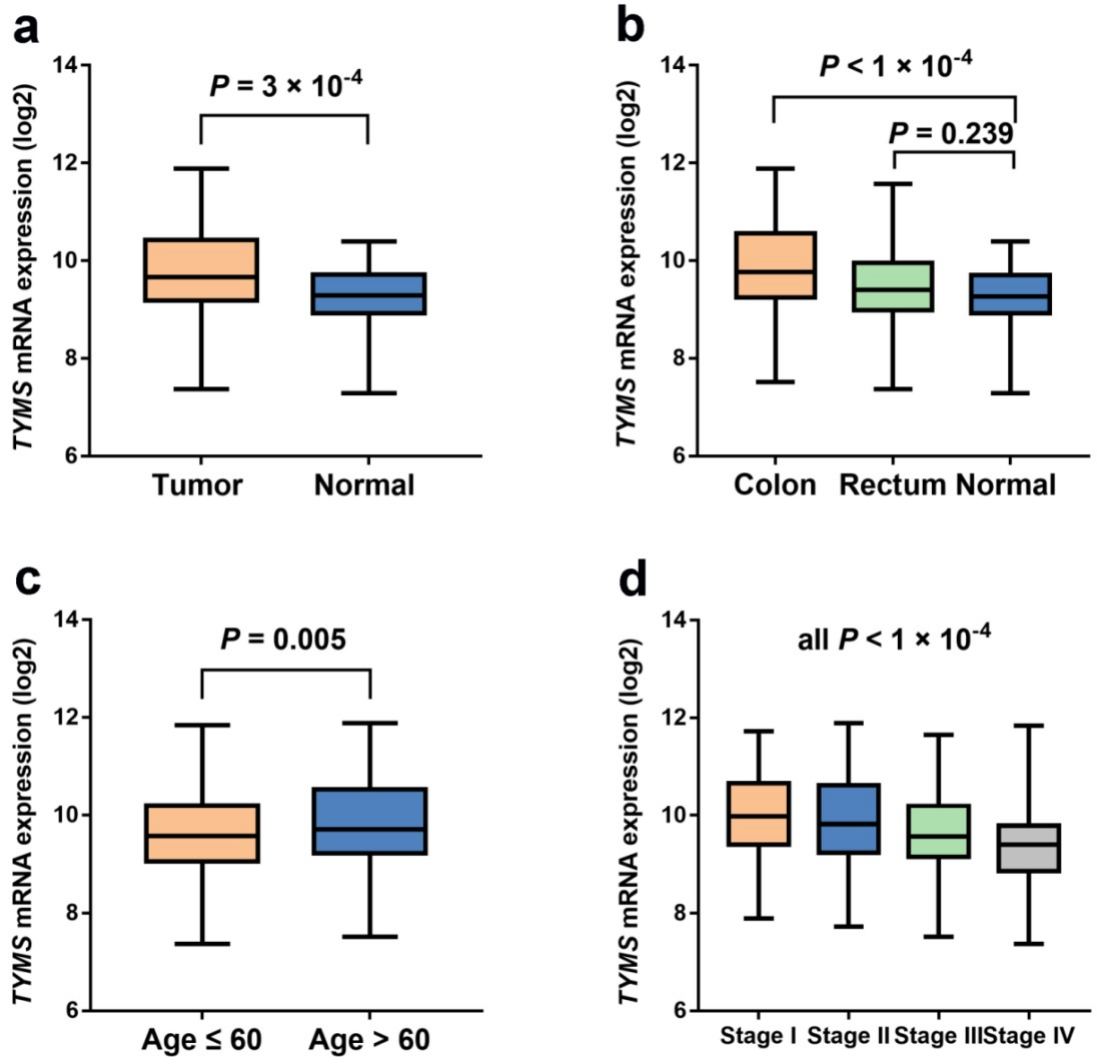
** The expression levels of *TYMS* in unpaired colorectal cancer and normal tissue samples from TCGA database.** (**a**) The expression levels of *TYMS* in unpaired colorectal cancer and normal tissue samples from TCGA database. (**b**) The expression levels of *TYMS* in unpaired colorectal cancer and normal tissue samples stratified by tumor site from TCGA database. (**c-d**) The expression levels of *TYMS* in colorectal cancer tissue samples stratified by age and tumor stage from TCGA database.

Our study indicated that the relationship between *TYMS* expression and OS of CRC patients was not statistically significant (*P* = 0.298) according to TCGA database (**Figure 2**). However, *TYMS* expression differences were significantly observed in our study. *TYMS* expression was increased in colorectal tumor tissues compared with adjacent tissues (*P* = 3.00 × 10^-4^) (**Figure 3**). Moreover, we assessed *TYMS* expression between colorectal tumor tissues and adjacent tissues based on tumor site. As a result, *TYMS* expression differences were only observed in colon cancer tissues and adjacent tissues (*P* < 1.00 × 10^-4^). Differential expression of *TYMS* was further analyzed in colorectal tumor tissues based on age, sex, CRC family history, BMI and tumor stage (**Figure 3 and Supplementary Figure S2**). Patients with younger age (*P* = 5.00 × 10^-3^) and advanced tumor stage (*P* < 1.00 × 10^-4^) were prone to have decreased expression of TYMS in our investigation.

## Discussion

In the present study, we assessed the relationship between SNPs in the folic acid metabolic pathway genes and survival, as well as responses of mCRC patients to first-line chemotherapy. We found that *TYMS* rs3786362 G allele was significantly correlated with reduced PFS, OS and DCR.

The folic acid metabolic pathway plays a vital role in the development of CRC. The abnormal growth of colon mucosa cells contributes to CRC development, which is affected by DNA repair genes, oncogenes and tumor suppressor genes^16^. The folic acid family members are involved in the acceptation or reception of one-carbon units, thereby promoting pyrimidine and purine synthesis and various methylation reactions 17. One carbon derives from serine and then transfers to tetrahydrofolate (THF), a critical metabolite in the folic acid metabolic pathway. Then glycine and 5, 10-methylenetetrahydrofolate (5, 10-MTHF) are generated by serine hydroxymethyltransferase (*SHMT*). The conversion of dUMP to dTMP is catalyzed by* TYMS*, providing precursors for DNA synthesis. This progress is carried out with the availability of 5, 10-methylene-THF. The generated metabolite of this reaction is dihydrofolate (DHF), which is converted into THF with the help of dihydrofolate reductase (*DHFR*) 18.

Folic acid metabolism, known as one-carbon metabolism, also regulates DNA methylation reactions through the reduction of 5, 10-MTHF to 5-MTHF, which was mediated by the activity of *MTHFR* 19. Methionine synthase (*MTR*) plays an essential role in converting 5-MTHF and homocysteine to THF and methionine. S-adenosyl methionine (SAM) generated by the methionine metabolism serves as a methyl donor to DNA, RNA and phospholipids. SAM can also inhibit the activity of *MTHFR*, which regulates its cell expression. The folate coenzymes are activated to DNA synthesis with increasing expression of SAM. However, folate deficiency can depress the production of SAM and cancel the inhibition of *MTHFR*, resulting in a decline in nucleotide synthesis 20.

*TYMS* helps to convert dUMP and DHF to dTMP and 5, 10-methylene-THF in the folic acid metabolic pathway. Regulation of the *TYMS* reaction is essential for DNA synthesis due to its vital role in the pathway. *TYMS* impairment might be critical for point mutation formation, uracil misincorporation into DNA and cancerogenesis 21. *TYMS* is a treatment target of 5-FU. FdUMP, the metabolite of 5-FU, combines *TYMS* to form a ternary complex, thereby inhibiting the normal function of *TYMS* with the help of 5, 10-MTHF. Therefore, the genetic variants in folic acid metabolic pathway could influence the survival of CRC patients as a deficiency in cell folates leads to point mutations, aberrant DNA methylation, increased frequency of micronuclei and chromosome breakage 22. In 2014, FH. *et.al* 23 published a paper in which the relationship between low folate status and various cancers such as prostate, breast, and colorectal cancer was reported. Our study suggested that *TYMS* rs3786362 G allele was related to reduced PFS, OS and DCR of mCRC patients. We hypothesized that rs3786362 G allele in *TYMS* might be a potential predictive biomarker for survival and responses to first-line chemotherapy of mCRC patients by affecting the normal function of *TYMS* gene and folic acid metabolism. The mechanisms underlying the potential predictive ability of *TYMS* rs3786362 G allele may be its biological functions in disturbing the conversion of dUMP and DHF to dTMP and 5, 10-methylene-THF, thereby resulting in abnormal DNA synthesis.

The mutations in *TYMS* (g.657795_657826del, c.53_84del and p.Gln18Argfs*42) were reported to connect with survival of CRC 24. A meta-analysis conducted by Jennings BA *et.al* 25 suggested that *TYMS* rs45445694 was associated with the reduced protein expression and improved clinical benefit from 5-FU. However, inconsistent results were found in other investigations 26. Therefore, we analyzed the correlation between genetic variants in 15 key genes in folic acid metabolic pathway and survival, as well as responses to chemotherapy of mCRC patients. Three models (the additive, dominant, and recessive models) were applied to further investigate the interested SNPs. It was found that *TYMS* rs3786362 was associated with PFS, OS and DCR only in the additive and dominant models. It may be due to the the low mutation frequency of *TYMS* rs3786362 in Chinese population. According to TCGA database, *TYMS* expression was statistically higher in CRC tissues compared to adjacent tissues, indicating the positive effects of *TYMS* in cancer development. We observed the positive relationship between *TYMS* expression and CRC survival, but it was not statistically significant. As our study employing the TCGA database only included the American and European populations, more patients and follow-ups are needed to validate the correlation between *TYMS* expression and CRC survival in Chinese population.

We conducted the stratification analysis in subgroups of sex, age, tumor site, tumor grade, number of metastatic organism, drinking status, smoking status, family history, Dukes stage and chemotherapy regimens. It was found that *TYMS* rs3786362 AG/GG genotypes indicated shorter PFS time for recipients of oxaliplatin-based chemotherapies, and shorter OS time for recipients of irinotecan-based chemotherapies. A previous meta-analysis including 7 clinical studies revealed that the response rate of patients treated with oxaliplatin-based chemotherapies was higher than those in irinotecan group. In addition, the OS time was longer in oxaliplatin group compared with that in irinotecan group 27. This contradictory result may be due to different sensitivity and tolerance of chemotherapy for individuals. Larger cohort studies are needed to confirm the relationship between *TYMS* rs3786362 and sensitivity of different chemotherapy regimens for mCRC patients.

There were some inherent limitations in our study. Firstly, clinical characteristics of patients may have introduced bias to our results due to the relatively small sample size. Therefore, large populations are warranted to confirm our findings. Secondly, we tried to conduct the eQTL analysis based on GTEx and TCGA database, but no relevant results were observed as the mutation frequency of rs3786362 was less than 0.01 in Western population. Therefore, the eQTL analysis should be further carried out in our study in Chinese population. Thirdly, no direct biology experiments were performed *in vitro* or *in vivo* for additional validations in our study.

In summary, our study provided evidence that the genetic variants in the folic acid metabolic pathway genes were associated with outcomes of mCRC patients undergoing first-line chemotherapy. Our study suggested that the *TYMS* rs3786362 G allele might be a potential predictive biomarker for reduced PFS, OS and DCR of mCRC patients receiving first-line chemotherapy, which might be the scientific foundation to predict survival and first-line chemotherapy efficiency of mCRC patients in the future.

## Supplementary Material

Supplementary figures and tables.

## Figures and Tables

**Table 1:** Association of four significant SNPs with mCRC outcomes

SNP	Gene	Chromosome	Position	Allele	*P*_(HWE)_	PFS			DCR	
						Adjusted HR (95%CI)	*P*	*P*_FDR_	Adjusted OR (95%CI)	*P*
rs369803	*FOLH1*	11	49174367	C>T	0.669	**0.68 (0.51-0.89)**	**0.006**	**0.100**	0.98 (0.55-1.68)	0.944
rs4795436	*SLC46A1*	17	26729428	C>T	0.873	1.28 (1.02-1.61)	0.032	0.280	-	-
rs3786362	*TYMS*	18	662247	G>A	0.434	**1.43 (1.12-1.82)**	**0.004**	**0.100**	**1.97 (1.19-3.27)**	**0.008**
rs10432965	*FTCD*	21	47557222	A>G	0.190	0.81 (0.66-0.98)	0.027	0.280	-	-

HR: hazard ratio; CI: confidence interval; *P*: for additive model adjusted for sex and age in Cox and logistic regression models; *P*_FDR_: for additive model adjusted for sex and age in Cox regression model after the false discovery rate (FDR) correction.

**Table 2:** Association of *TYMS* rs3786362 and mCRC survival

	PFS						OS					
Genotyping	Cases	No. of progression (%)	HR^a^ (95%CI)	*P*^a^	HR^b^ (95%CI)	*P*^b^	Cases	No. of death (%)	HR^a^ (95%CI)	P^a^	HR^b^ (95%CI)	P^b^
AA	221	151 (68.3)	1.00		1.00		221	97 (43.9)	1.00		1.00	
AG	97	76 (78.4)	**1.65 (1.24-2.19)**	**0.001**	**1.62 (1.21-2.15)**	**0.001**	97	50 (51.5)	**1.54 (1.09-2.18)**	**0.014**	**1.58 (1.11-2.24)**	**0.010**
GG	7	4 (57.1)	0.96 (0.35-2.63)	0.943	0.88 (0.32-2.42)	0.807	7	3 (42.9)	1.13 (0.36-3.58)	0.839	1.15 (0.37-3.65)	0.812
Additive model			**1.43 (1.12-1.82)**	**0.004**	**1.37 (1.08-1.75)**	**0.011**			**1.43 (1.03-1.85)**	**0.019**	**1.40 (1.04-1.88)**	**0.025**
Dominant model			**1.61 (1.20-2.10)**	**0.001**	**1.55 (1.17-2.05)**	**0.002**			**1.52 (1.08-2.14)**	**0.015**	**1.55 (1.10-2.18)**	**0.012**
Recessive model			0.83 (0.31-2.26)	0.720	0.76 (0.28-2.09)	0.601			1.00 (0.32-3.15)	0.997	1.02 (0.32-3.21)	0.977

PFS: progression-free survival; OS: overall survival; HR: hazard ratio; CI: confidence interval; a: adjusted for sex and age in Cox regression model; b: adjusted for sex, age, tumor site and chemotherapy in Cox regression model.

**Table 3:** Association of *TYMS* rs3786362 and responses to chemotherapy in mCRC patients

Genotyping	DCR					
	Cases	No. of PD (%)	OR^a^ (95%CI)	*P*^a^	OR^b^ (95%CI)	*P*^b^
AA	221	34 (15.4)	1		1	
AG	97	31 (32.0)	**2.47 (1.40-4.36)**	**0.002**	**2.52 (1.42-4.48)**	**0.001**
GG	7	1 (14.3)	1.04 (0.12-9.26)	0.970	1.25 (0.14-11.42)	0.844
Additive model			**1.97 (1.19-3.27)**	**0.008**	**2.07 (1.24-3.44)**	**0.005**
Dominant model			**2.37 (1.35-4.15)**	**0.003**	**2.44 (1.38-4.30)**	**0.002**
Recessive model			0.75 (0.09-6.56)	0.794	0.90 (0.10-8.15)	0.928

PD: progress disease; OR: odds ratio; CI: confidence interval; a: adjusted for sex and age in logistic regression models; b: adjusted for sex, age, tumor site and chemotherapy in logistic regression models.

**Table 4:** Stratification analysis for the association between rs3786362 and mCRC survival in dominant model

Variable	*TYMS* rs3786362		PFS		OS	
	No. of (AG+GG) (%)	No. of AA (%)	HR (95% CI)	*P*	HR (95% CI)	*P*
**Sex**						
Male	70 (34.1)	135 (65.9)	1.34 (0.96-1.88)	0.088	**1.67 (1.11-2.52)**	**0.013**
Female	34 (28.3)	86 (71.7)	**2.09 (1.26-3.44)**	**0.004**	1.33 (0.70-2.53)	0.378
**Age**						
≤60	60 (34.3)	115 (65.7)	**1.69 (1.17-2.45)**	**0.005**	**1.70 (1.07-2.70)**	**0.024**
>60	44 (29.3)	106 (70.7)	1.45 (0.94-2.32)	0.089	1.39 (0.83-2.33)	0.213
**Tumor site**						
Colon	64 (33.0)	130 (67.0)	**1.82 (1.25-2.66)**	**0.002**	1.26 (0.80-1.98)	0.314
Rectum	40 (30.5)	91 (69.5)	1.27 (0.83-1.95)	0.272	**2.13 (1.26-3.60)**	**0.005**
**Tumor grade**						
Well + Moderate	78 (30.6)	177 (69.4)	**1.77 (1.28-2.44)**	**0.001**	**1.71 (1.15-2.55)**	**0.008**
Poor	26 (37.1)	44 (62.9)	1.20 (0.62-2.32)	0.584	0.96 (0.45-2.06)	0.916
**Number of metastatic organism**						
≤2	86 (31.5)	187 (68.5)	**1.80 (1.32-2.45)**	**<0.001**	**1.69 (1.15-2.48)**	**0.007**
>2	18 (34.6)	34 (65.4)	0.97 (0.45-2.11)	0.947	1.03 (0.47-2.27)	0.945
**Drinking status**						
Yes	70 (31.0)	156 (69.0)	**1.63 (1.15-2.30)**	**0.006**	1.22 (0.80-1.86)	0.355
No	34 (34.3)	65 (65.7)	1.46 (0.88-2.42)	0.141	**2.86 (1.51-5.42)**	**0.001**
**Smoking status**						
Yes	67 (31.5)	146 (68.5)	1.32 (0.92-1.91)	0.131	1.27 (0.81-1.98)	0.301
No	37 (33.0)	75 (67.0)	**2.22 (1.32-3.60)**	**0.001**	**2.47 (1.38-4.43)**	**0.002**
**Family history**						
Yes	21 (37.5)	35 (62.5)	1.39 (0.65-2.96)	0.396	1.82 (0.85-3.90)	0.126
No	83 (30.9)	186 (69.1)	**1.60 (1.17-2.22)**	**0.003**	1.43 (0.96-2.12)	0.077
**Chemotherapy**						
Oxaliplatin	61 (32.4)	127 (67.6)	**1.97 (1.33-2.92)**	**0.001**	1.43 (0.90-2.26)	0.130
Irinotecan	43 (31.4)	94 (68.6)	1.22 (0.81-1.83)	0.351	**1.79 (1.07-3.01)**	**0.027**

PFS: progression-free survival; OS: overall survival; HR: hazard ratio; CI: confidence interval; P: adjusted for sex, age, tumor site and chemotherapy in Cox regression model.
