# Supplementary material for: Genetic variants in the Folic acid Metabolic Pathway Genes predict outcomes of metastatic Colorectal Cancer patients receiving first-line Chemotherapy
Source: J Cancer. 2020 Sep 21;11(22):6507–15. doi: 10.7150/jca.44580 (PMC7545690; doi:10.7150/jca.44580)
Supplement: Supplementary file 1 — Supplementary figures and tables. [file jcav11p6507s1.pdf]

Supplementary Table 1. Clinical characteristics and their association with overall survival of mCRC patients.

Variable	Cases	No. of deaths (%)	Univariate analysis		Multivariate analysis	
			**HR[a] (95%CI)**	***P*[a]**	**HR[b] (95%CI)**	***P*[b]**
**Sex**						
Male	205	101 (49.3)	1.00		1.00	
Female	120	49 (40.8)	0.89 (0.63–1.26)	0.519	0.90 (0.64–1.27)	0.549
**Age**						
≤60	175	78 (44.6)	1.00		1.00	
>60	150	72 (48.0)	1.15 (0.83–1.58)	0.401	1.14 (0.83–1.58)	0.423
**Tumor site**						
Colon	194	85 (43.8)	1.00		1.00	
Rectum	131	65 (49.6)	1.08 (0.78–1.49)	0.651	1.08 (0.78–1.49)	0.657
**Tumor grade**						
Well + Moderate	255	112 (43.9)	1.00		1.00	
Poor	70	38 (54.3)	1.39 (0.96–2.00)	0.084	1.44 (0.99–2.10)	0.057
**Number of metastatic organism**						
≤2	237	119 (43.6)	1.00		1.00	
>2	52	31 (59.6)	1.46 (0.98–2.17)	0.060	1.48 (0.99–2.20)	0.054
**Drinking status**						
Yes	226	106 (46.9)	1.00		1.00	
No	99	44 (44.4)	0.91 (0.64–1.30)	0.612	0.84 (0.57–1.24)	0.384
**Smoking status**						
Yes	213	92 (43.2)	1.00		1.00	
No	112	58 (51.8)	1.26 (0.90–1.75)	0.174	1.25 (0.86–1.80)	0.242
**Family history**						
Yes	56	30 (53.6)	1.00		1.00	
No	269	120 (44.6)	0.88 (0.59–1.32)	0.548	0.88 (0.59–1.31)	0.517
**Dukes stage**						
C	23	7 (30.4)	1.00		1.00	
D	302	143 (47.4)	0.67 (0.31–1.45)	0.308	0.66 (0.30–1.43)	0.288
**Chemotherapy**						
Oxaliplatin	188	81 (43.1)	1.00		1.00	
Irinotecan	137	69 (50.4)	1.21 (0.88–1.67)	0.242	1.23 (0.89–1.70)	0.219

HR: hazard ratio; CI: confidence interval.

a: unadjusted in logistic regression model.

b: adjusted for sex, age, tumor site and chemotherapy in logistic regression model.

Supplementary Table 2. List of the selected 15 genes in folic acid metabolic pathway.

Gene	Description	Chromosome	Start	End
MTR	5-methyltetrahydrofolate-homocysteine methyltransferase	1	236958581	237067281
MTHFR	Methylenetetrahydrofolate reductase	1	11845787	11866160
ALDH1L1	Aldehyde dehydrogenase 1 family member L1	3	125822404	125900029
DHFR	Dihydrofolate reductase	5	79922045	79950800
MTRR	5-methyltetrahydrofolate-homocysteine methyltransferase reductase	5	7851299	7901237
FPGS	Folylpolyglutamate synthase	9	130565137	130576799
FOLH1	Folate hydrolase 1	11	49168187	49230222
MTHFD1	Methylenetetrahydrofolate dehydrogenase, cyclohydrolase and formyltetrahydrofolate synthetase 1	14	64854754	64926725
MTHFS	Methenyltetrahydrofolate synthetase	15	80135889	80189627
SHMT1	Serine hydroxymethyltransferase 1	17	18231187	18266856
SLC46A1	Solute carrier family 46 member 1	17	26721661	26733230
TYMS	Thymidylate synthetase	18	657590	673499
CBS	Cystathionine-beta-synthase	21	44473301	44496472
FTCD	Formimidoyltransferase cyclodeaminase	21	47556065	47575499
SLC19A1	Solute carrier family 19 member 1	21	46934629	46983045

Supplementary Table 3. ***In silico*** **analysis for SNPs function annotation.**

SNP	Gene	Chromosome	Position	Allele	MAF	Regulome DB Score	HaploReg
rs9651118	*MTHFR*	1	11862214	C>T	0.34	3a	DNAse, motifs changed, selected eQTL hits
rs1801133	*MTHFR*	1	11856378	A>G	0.42	4	DNAse, proteins bound, motifs changed, selected eQTL hits
rs12022198	*MTR*	1	236989578	C>T	0.19	5	motifs changed
rs4646760	*ALDH1L1*	3	125822871	C>T	0.17	4	Proteins bound, motifs changed, selected eQTL hits
rs4646759	*ALDH1L1*	3	125822946	C>G	0.11	4	Proteins bound, motifs changed, selected eQTL hits
rs3772431	*ALDH1L1*	3	125826914	C>T	0.47	2b	DNAse, motifs changed, selected eQTL hits
rs162024	*MTRR*	5	7860404	T>G	0.37	1f	Selected eQTL hits
rs4277880	*MTRR*	5	7857424	T>C	0.30	1f	DNAse, motifs changed, selected eQTL hits
rs6555501	*MTRR*	5	7879983	C>T	0.34	1f	motifs changed, selected eQTL hits
rs326124	*MTRR*	5	7877178	A>G	0.35	1b	DNAse, proteins bound, motifs changed, selected eQTL hits
rs161869	*MTRR*	5	7877831	T>C	0.33	1f	motifs changed, selected eQTL hits
rs162040	*MTRR*	5	7887478	C>A	0.36	1b	DNAse, proteins bound, motifs changed, selected eQTL hits
rs326123	*MTRR*	5	7876861	G>A	0.48	1f	motifs changed, selected eQTL hits
rs3797191	*MTRR*	5	7877491	G>A	0.16	5	motifs changed, selected eQTL hits
rs10987742	*FPGS*	9	130565877	T>C	0.30	4	DNAse, proteins bound, motifs changed
rs10106	*FPGS*	9	130576075	T>C	0.33	2b	DNAse, motifs changed, selected eQTL hits
rs369803	*FOLH1*	11	49174367	C>T	0.16	N	Selected eQTL hits
rs35020344	*MTHFD1*	14	64855425	G>A	0.29	4	DNAse, proteins bound, motifs changed, selected eQTL hits
rs12434608	*MTHFD1*	14	64859293	G>C	0.41	3a	DNAse, proteins bound, motifs changed, selected eQTL hits
rs3783733	*MTHFD1*	14	64855721	G>A	0.11	4	DNAse, motifs changed
rs685487	*MTHFS*	15	80136129	G>A	0.45	1f	motifs changed, selected eQTL hits
rs4795436	*SLC46A1*	17	26729428	C>T	0.22	2b	DNAse, proteins bound, motifs changed
rs1128162	*SLC46A1*	17	26721791	C>A	0.26	4	DNAse, proteins bound, motifs changed, selected eQTL hits
rs2244500	*TYMS*	18	661005	A>G	0.34	1f	motifs changed, selected eQTL hits
rs1004474	*TYMS*	18	660383	G>A	0.44	1f	Selected eQTL hits
rs75075985	*TYMS*	18	660367	A>T	0.22	2b	motifs changed
rs3786362	*TYMS*	18	662247	G>A	0.17	2b	motifs changed
rs2853741	*TYMS*	18	657352	C>T	0.49	4	DNAse, proteins bound, motifs changed, selected eQTL hits
rs2124459	*CBS*	21	44475714	T>C	0.25	1b	DNAse, motifs changed, selected eQTL hits
rs1788466	*CBS*	21	44483773	A>G	0.18	4	DNAse, motifs changed, selected eQTL hits
rs234702	*CBS*	21	44477543	G>C	0.16	4	DNAse, motifs changed, selected eQTL hits
rs706209	*CBS*	21	44473425	G>A	0.35	1f	DNAse, proteins bound, selected eQTL hits
rs10432965	*FTCD*	21	47557222	A>G	0.37	3a	DNAse, proteins bound, motifs changed
rs2277821	*FTCD*	21	47575470	A>G	0.37	2b	DNAse, proteins bound
rs4818789	*SLC19A1*	21	46948827	G>T	0.21	4	DNAse, motifs changed

MAF: minor allele frequency.

Supplementary Table 4. Associations between all the significant SNPs and outcomes of mCRC patients.

SNP	Gene	Chromosome	Position	Allele	$P_{(HWE)}$	PFS		
						**Adjusted HR (95%CI)**	***P***	P_{FDR}
rs9651118	*MTHFR*	1	11862214	C>T	0.903	0.88(0.72-1.06)	0.185	0.500
rs1801133	*MTHFR*	1	11856378	A>G	0.210	1.06(0.89-1.26)	0.534	0.860
rs12022198	*MTR*	1	236989578	C>T	0.858	1.21(0.95-1.55)	0.128	0.410
rs4646760	*ALDH1L1*	3	125822871	C>T	0.566	0.91(0.72-1.17)	0.469	0.860
rs4646759	*ALDH1L1*	3	125822946	C>G	0.400	1.10(0.81-1.48)	0.542	0.860
rs3772431	*ALDH1L1*	3	125826914	C>T	0.824	1.01(0.85-1.21)	0.875	0.950
rs162024	*MTRR*	5	7860404	T>G	0.032	1.06(0.88-1.28)	0.524	0.860
rs4277880	*MTRR*	5	7857424	T>C	0.183	0.98(0.81-1.18)	0.830	0.950
rs6555501	*MTRR*	5	7879983	C>T	0.623	1.10(0.91-1.33)	0.328	0.720
rs326124	*MTRR*	5	7877178	A>G	0.396	0.86(0.71-1.03)	0.107	0.400
rs161869	*MTRR*	5	7877831	T>C	1.000	1.09(0.90-1.31)	0.395	0.810
rs162040	*MTRR*	5	7887478	C>A	0.469	0.90(0.75-1.09)	0.285	0.670
rs326123	*MTRR*	5	7876861	G>A	0.375	0.87(0.73-1.03)	0.113	0.400
rs3797191	*MTRR*	5	7877491	G>A	0.143	1.03(0.81-1.13)	0.792	0.950
rs10987742	*FPGS*	9	130565877	T>C	0.430	0.98(0.80-1.20)	0.856	0.950
rs10106	*FPGS*	9	130576075	T>C	1.000	0.98(0.80-1.20)	0.850	0.950
rs369803	*FOLH1*	11	49174367	C>T	0.669	**0.68(0.51-0.89)**	**0.006**	**0.100**
rs35020344	*MTHFD1*	14	64855425	G>A	0.688	1.04(0.85-1.27)	0.699	0.950
rs12434608	*MTHFD1*	14	64859293	G>C	0.205	1.01(0.84-1.22)	0.895	0.950
rs3783733	*MTHFD1*	14	64855721	G>A	1.000	0.95(0.70-1.28)	0.717	0.950
rs685487	*MTHFS*	15	80136129	G>A	0.435	1.17(0.99-1.39)	0.069	0.370
rs4795436	*SLC46A1*	17	26729428	C>T	0.873	**1.28(1.02-1.61)**	**0.032**	**0.280**
rs1128162	*SLC46A1*	17	26721791	C>A	0.671	1.12(0.91-1.38)	0.282	0.670
rs2244500	*TYMS*	18	661005	A>G	0.902	0.84(0.69-1.02)	0.083	0.370
rs1004474	*TYMS*	18	660383	G>A	0.312	1.20(0.99-1.47)	0.070	0.370
rs75075985	*TYMS*	18	660367	A>T	0.200	0.99(0.79-1.24)	0.921	0.950
rs3786362	*TYMS*	18	662247	G>A	0.434	**1.43(1.12-1.82)**	**0.004**	**0.100**
rs2853741	*TYMS*	18	657352	C>T	0.375	0.88(0.73-1.05)	0.149	0.440
rs2124459	*CBS*	21	44475714	T>C	0.140	1.00(0.81-1.22)	0.964	0.960
rs1788466	*CBS*	21	44483773	A>G	0.457	0.97(0.76-1.23)	0.784	0.950
rs234702	*CBS*	21	44477543	G>C	0.219	1.02(0.77-1.35)	0.911	0.950
rs706209	*CBS*	21	44473425	G>A	0.904	0.97(0.80-1.18)	0.784	0.950
rs10432965	*FTCD*	21	47557222	A>G	0.190	**0.81(0.66-0.98)**	**0.027**	**0.280**
rs2277821	*FTCD*	21	47575470	A>G	0.339	0.94(0.78-1.14)	0.517	0.860
rs4818789	*SLC19A1*	21	46948827	G>T	0.509	1.22(0.97-1.52)	0.086	0.370

HR: hazard ratio; CI: confidence interval.

P: for additive model adjusted for sex and age in logistic regression model.

P_{FDR}: for additive model adjusted for sex and age in logistic regression model after the false discovery rate (FDR) correction.

Figure Legends

Supplementary Figure 1. Key genes in the folic acid metabolic pathway. *DHFR*: dihydrofolate reductase; *FOLH1*: folate hydrolase 1; *FPGS*: folylpolyglutamate synthase; *MTHFD1*: methylenetetrahydrofolate dehydrogenase, cyclohydrolase and formyltetrahydrofolate synthetase 1; *MTHFR*: methylenetetrahydrofolate reductase; *MTR*: 5-methyltetrahydrofolate-homocysteine methyltransferase; *MTRR*: 5-methyltetrahydrofolate-homocysteine methyltransferase reductase; *SHMT1*: serine hydroxymethyltransferase 1; *SLC19A1*: solute carrier family 19 member 1; *SLC46A1*: solute carrier family 46 member 1; *TYMS*: thymidylate synthetase; *CBS*: cystathionine-beta-synthase; *FTCD*: formimidoyltransferase cyclodeaminase; *ALDH1L1*: cytosolic 10-formyltetrahydrofolate dehydrogenase; *MTHFS*: 5-formyltetrahydrofolate cyclo-ligase.

Supplementary Figure 2. The mRNA expression levels of *TYMS* in colorectal cancer tissue samples stratified by sex, family history and BMI from TCGA database. BMI: body mass index.

Supplementary Figure 1. Key genes in the folic acid metabolic pathway.

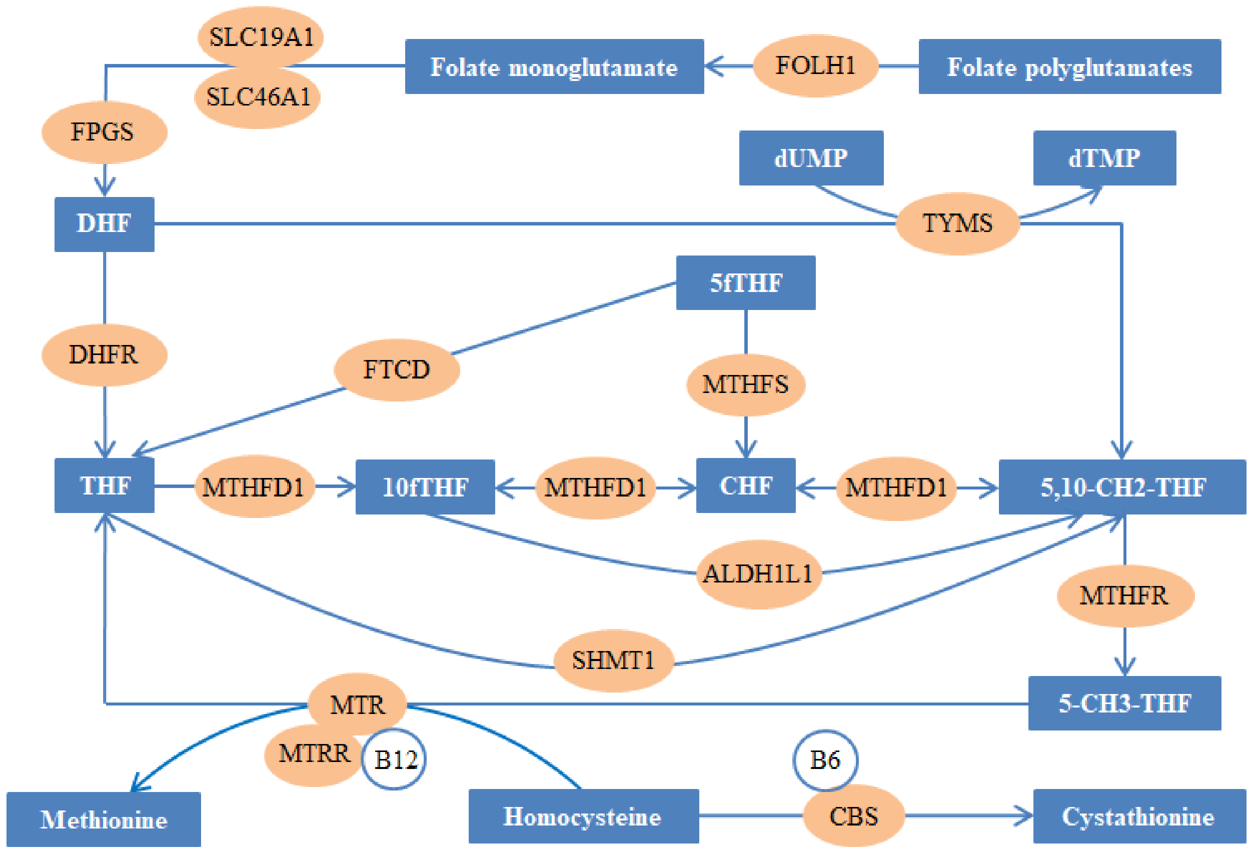

DHFR: dihydrofolate reductase; *FOLH1*: folate hydrolase 1; *FPGS*: folylpolyglutamate synthase; *MTHFD1*: methylenetetrahydrofolate dehydrogenase, cyclohydrolase and formyltetrahydrofolate synthetase 1; *MTHFR*: methylenetetrahydrofolate reductase; *MTR*: 5-methyltetrahydrofolate-homocysteine methyltransferase; *MTRR*: 5-methyltetrahydrofolate-homocysteine methyltransferase reductase; *SHMT1*: serine hydroxymethyltransferase 1; *SLC19A1*: solute carrier family 19 member 1; *SLC46A1*: solute carrier family 46 member 1; *TYMS*: thymidylate synthetase; *CBS*: cystathionine-beta-synthase; *FTCD*: formimidoyltransferase cyclodeaminase; *ALDH1L1*: cytosolic 10-formyltetrahydrofolate dehydrogenase; *MTHFS*: 5-formyltetrahydrofolate cyclo-ligase.

Supplementary Figure 2. The mRNA expression levels of *TYMS* in colorectal cancer tissue samples stratified by sex, colorectal cancer family history and BMI from TCGA database.

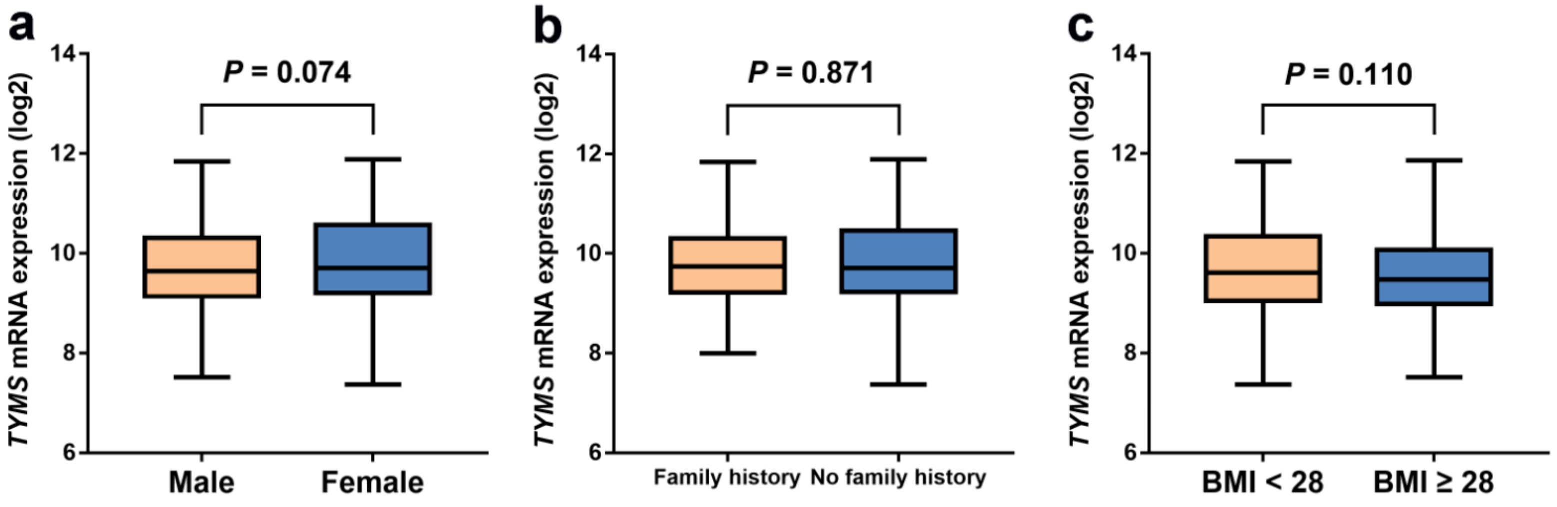

BMI: body mass index.
